# Working on and with Relationships: Relational Work and Spatial Understandings of Good Care in Community Mental Healthcare in Trieste

**DOI:** 10.1007/s11013-020-09672-8

**Published:** 2020-04-03

**Authors:** Christien Muusse, Hans Kroon, Cornelis L. Mulder, Jeannette Pols

**Affiliations:** 1 Trimbos Institute, Netherlands Institute of Mental Health and Addiction, Utrecht, The Netherlands; 2 Section of Medical Ethics, Department of General Practice, UMC Amsterdam, Amsterdam, The Netherlands; 3 Tranzo, School of Social and Behavioural Sciences, Tilburg University, Tilburg, The Netherlands; 4 Erasmus MC, Rotterdam, The Netherlands; 5 Parnassia Psychiatric Institute, The Hague, The Netherlands; 6 Department of Anthropology, University of Amsterdam, Amsterdam, The Netherlands

**Keywords:** Deinstitutionalization, Empirical ethics, Community mental healthcare, Spatial metaphors, Care collectives

## Abstract

Deinstitutionalization is often described as an organizational shift of moving care from the psychiatric hospital towards the community. This paper analyses deinstitutionalization as a daily care practice by adopting an empirical ethics approach instead. Deinstitutionalization of mental healthcare is seen as an important way of improving the quality of lives of people suffering from severe mental illness. But how is this done in practice and which different goods are strived for by those involved? We examine these questions by giving an ethnographic description of community mental health care in Trieste, a city that underwent a radical process of deinstitutionalization in the 1970s. We show that paying attention to the spatial metaphors used in daily care direct us to different notions of good care in which relationships are central. Addressing the question of how daily care practices of mental healthcare outside the hospital may be constituted and the importance of spatial metaphors used may inform other practices that want to shape community mental health care.

## Introduction

Along the coastline of Trieste, on the western side of the city, a busy street runs into the center. Along it lie some bars, an expensive hotel, a restaurant and a church. A bit further down is a popular place where *Triestiani* go for a swim in the sea on warm days. One building is an old, yellowish villa surrounded by a garden. Go through the porch, and you will find a large table in the garden where people sit chatting and smoking cigarettes. The house itself is a two-floor building, with a small balcony just above the entrance. You are now entering Trieste’s first community mental health center, a place with an interesting history.

Since the 1970s, Trieste, a city high in Italy’s northeast, has been the stage for visitors from all over the world inspired by the way community mental health is done there. The so-called ‘Trieste model’ of mental health care that has been in constant development since then, was inspired by the ideas of Franco Basaglia (1924–1980), a psychiatrist and leading figure in Italy’s democratic psychiatry movement. Basaglia proclaimed that the person, rather than the mental health disorder, should be placed at the center of the mental healthcare system (Portacolone et al. 2015). On the basis of his philosophy, the psychiatric hospital was closed and a radical shift started towards care in the community.

While the community mental health system that has developed since has changed over time, it has maintained its basic principles: no closed doors, no seclusion and a minimum of restraint. The approach in Trieste has also attracted a lot of attention from professional mental health care workers in and outside Italy, raising questions about what one might term the ‘active ingredients’ of deinstitutionalization in Italy (Barbui and Tansella 2008), and whether the ‘Trieste model’ can be translated to other countries (Portacolone et al. 2015).

### Shift from Organization to Daily Practice

In this article we want to explore what daily care practices in Trieste can teach us about deinstitutionalization, how care in the community can be shaped, and what makes it *good* care. We adopt an empirical ethics approach to shift the focus from a sole organizational perspective towards deinstitutionalization—or the ‘bricks and mortar’ as Chow and Priebe (2013) called it—towards the daily practice of care, the values that come matter and how these values relate to notions of space. An empirical ethics approach is useful to make this shift due to its focus on daily practices and its focus on how these practices entail different notions of what is good care (Willems and Pols 2010). What is ‘good’ is not defined beforehand, but depends on what actors in a specific care practice strive for or bring about as a good practice (Pols 2014). So the shift we make in this article is from how deinstitutionalization is *organized* to how it is *practiced in everyday care.*^1^ We examine how mental health care in the community can be shaped, and what makes it good care or better care—questions that are relevant to other countries where deinstitutionalization is under debate. Due to its radical ideas and its shaping of the process of deinstitutionalization towards care in the community, mental health care in Trieste is an interesting case from which others can learn (Burti 2001).

Our argument is twofold. First, by drawing on ethnographic fieldwork, we explore the ‘the goods’ the actors in care situations in Trieste strive for, and the ‘bads’ they try to avoid. We demonstrate how professionals build and maintain a social network around people with mental health problems by working *with* and *on* relationships. This is crucial to understanding how the practice of community mental healthcare in Trieste constitutes *good* care. Drawing on the work of Myriam Winance, we argue that it is necessary to widen our understanding of a care relationship, not just as a relationship between a care professional and a patient, but as a broader care collective in which people and other nonsocial actors—routines, environments and things—interrelate and together make up care practices. We are particularly interested in the role, understanding and practices of mental health care professionals, and in how they deployed to bring about something ‘good’.

Second, we take this empirical ethics approach one step further by unraveling the different spatial metaphors that are used to describe different notions ‘good care’. We argue the importance of spatial metaphors to care, showing how spatial understandings of care shape thinking about relationships, and orient actors towards particular forms of good care. We identify two different spatial metaphors in care practice in Trieste that relate to different scenarios for shaping good care. In practice, these can lead to frictions. Paying attention to these frictions helps to understand care as a continuous process of negotiating on the ‘good’.

## Background

### Basaglia’s Legacy

In the 1960s, first in Gorizia and then in Trieste, Franco Basaglia, a young psychiatrist from Venice, started to experiment with new forms of psychiatry. Inspired by thinkers such as Foucault, Fanon and Goffman, Basaglia saw a mental hospital as a ‘total institution’ that deprived people of their identity and turned them into ‘non-humans’. Goffman (1968) describes how the life of patients in a psychiatric hospital was dictated by the routine of the institution, and how they were isolated from the rest of society. This led to a ‘mortification of the self’, a process in which an individual was stripped of any former roles, and instead took on a purely institutional role: that of a patient (Chow and Priebe 2013).

Around Basaglia emerged a movement that, in practice but also in text, film and photography (Foot, 2015b), called for the reform of psychiatry. It was in Gorizia, where Basaglia as a director started to introduce reforms, first by improving the conditions of patients still in hospital: ending restraint, reducing electro-shock treatment, opening up wards, and destroying walls and fences (Foot 2014, 2015a). In 1971, after leaving Gorizia, Basaglia started as a director of the asylum in Trieste. It was there that he started to put his ideas into practice in a more radical way. The plan was no longer to reform mental hospitals from within, but to break them down entirely, as a way of breaking the power of the institution.

The start of the closure of the mental hospital in Trieste in the 1970s did not happen in a vacuum: Since World War II, consensus had been growing on the need for a thorough change in mental health care. Central to this movement was a shared objective that the old asylum-based system of care should be replaced by new community-orientated approaches (Novella 2010; Shen and Snowden 2014; Bennett 1985). The movement in Trieste should thus be understood against the background of this historical context, which was characterized by the social, protest and civil rights movements of the so-called 1968 revolution. In this period, psychiatric institutions became the focus of political struggle and places of social experimentation (Henckes 2011). Naturally, the process of psychiatric reform varied from one country to another and had various influences: the development of a welfare state, changes in the status and roles of the medical profession, and the needs to reduce costs and contain risk (Henckes 2011; Chow, Ajaz and Priebe 2018). But even though reforms were context-bound, developments in Italy—and particularly in Trieste—were an inspiration for the processes of deinstitutionalization taking place in many other parts of the world. Law 180, which called for the closure of psychiatric hospitals throughout Italy, starting by ending new admissions in order to phase them out altogether, was seen as the first deinstitutionalization law and came into force in 1978 (Portacolone et al. 2015). Its actual implementation varied greatly between the various regions of Italy.^2^

### Trieste’s Mental Health System

The closure of Trieste’s San Giovanni Psychiatric Hospital initiated the building of a new system of mental health care in the community. The city’s mental health care services are organized on the principle of territorial responsibility, each of the four municipal districts having a Community Mental Health Center (CMHC) with responsibility for all those with mental health problems in his area. Each CMHC team consists of nurses, psychiatrists, psychologists, rehabilitation specialists and social workers. All nurses work on the reception, rotating on intake functions. People can call in or walk into seek help. Some of them are seeking help for themselves, others are their relatives, neighbors, or partner organizations. There is no waiting list and no need for a referral. The center is also the place where treatment and care are provided and where people can have lunch or just ‘hang out’ for some time during the day. Each CMHC team provides home visits. People also come to the center for therapeutic interventions and to collect medication. For people who need to stay overnight, there are a total of six beds in one-person or two-person rooms. They can receive visits without restrictions. Occasionally, relatives can stay overnight if necessary. On average, people stay 11 days (Mezzina 2014; Muusse and Van Rooijen 2015).

At night, two nurses are present to attend to those sleeping at the center. If someone needs psychiatric care after 8.00 p.m., she or he is referred to the crisis department at the general hospital, which has a small acute ward with six beds. The following morning, contact is made with the center in her or his city district. Here, the average stay is 3 days, though if diagnostic tests or hospital care are needed, some patients may stay longer. To further reduce the number of hospitalizations, this department has recently been experimenting with the organization of Intensive Home Treatment in cases of crisis. Neither the centers nor the hospital ward have seclusion facilities or closed doors: if compulsory care is necessary, a center will organize it by intensifying the care it provides (Mezzina 2014; Muusse and Van Rooijen 2015). This may mean, for instance, that mental healthcare workers, volunteers or family members accompany a service user during the day. Numbers of compulsory care are low compared to other countries, but also in comparison to other regions in Italy (Mezzina 2016).

As the centers are part of the local healthcare agency, they are also part of the public health service. They work with the prison, the police, community primary care teams (Health Care Districts e.g., for young, elderly and disabled people) and welfare organizations, which are one of the partners of the CMHC with whom they consult and share planning (Mezzina 2016). Various other services, including the rehabilitation services, are part of the community mental health department. The centers also work with services that are closely related to mental healthcare, such as self-help groups and family organizations. Of special interest here are the social cooperatives, in which people with and without a disability work together on an equal basis. Personal projects are usually developed in co-production with coops and associations and through personal healthcare budgets for complex needs (Ridente and Mezzina 2016).

## Methods

### Empirical Ethics and Spatial Metaphors

This study describes community mental health care in Trieste from an ethnographic perspective. It focuses particularly on care situations that are labeled as the onset of a psychiatric crisis, and what the actors involved perceive as ‘good care’ in these situations. To do this we approach care as it is enacted through everyday practices that include people, as well as things and technologies (Mol, Moser, and Pols 2010; Pols 2012, 2015). What is important in this approach to care ethics is the notion of the “relational interdependence of people as subjects that are caring and cared for” (Pols 2014:81). It takes account of the changing relationships between service users, caregivers, material conditions and others, unraveling their relationships and their *intra*-*normativity* through a focus on everyday care practices (ibid).

We are particularly interested in how ideas about good care are enacted in daily practice and how the people involved deal with the tensions that sometimes arise (Willems and Pols 2010). Rather than defining beforehand what this ‘good’ is, we use participant observation to study it in practice (Pols 2014). In many care situations it is not always apparent what is good and what is bad. What is ‘good’ can be complex and ambivalent; it depends on the situation and is not always easy to translate into practice (Mol, Moser, and Pols 2010).

In an extension of our empirical ethics approach, we argue that ideas about good care are often related to spatial metaphors. In this, we draw on the work of Mol and Law (1994), who distinguish between regional space and networks space^3^ as two different forms of spatiality. Regional space is the physical space, the boundary between here and there. In “regions […] objects are clustered together and boundaries are drawn around each cluster” (p. 643). Network space, in contrast, links objects in space through the relationships between them, rather than through a regional ordering (p. 649). One can be ‘within’ a network space by becoming part of this network, wherever one might be physically located. Internet networks are clear examples of this; they link people together, without them being in the same region. We will show not only how different notions of space are related to different scenarios for shaping good care, but also how there can be tensions between them. Paying attention to such tensions provides insight into the scenarios for shaping good care that are embedded in daily practices. More importantly, such attention can also help us to strengthen these practices.

### Fieldwork

The first author has been involved in studies on community mental healthcare in Trieste since 2014. The first study (in 2014) is included in the report “Freedom first”, which was based mostly on interviews, but also included some observational work (Muusse and Van Rooijen 2015).^4^ Having visited Trieste’s mental health care services several times since 2014, and two counter visits from care professionals from Trieste to the Netherlands, the idea emerged that it would be valuable to conduct in-depth research from an ethnographic perspective. The current research involved observations divided over three periods in 2017 at one of the city’s mental health centers. Although the first author has a basic understanding of Italian, to gain a detailed understanding of daily practice there, she worked with an interpreter who was familiar with mental health care.^5^ Together they observed the daily routines of the CMHC referred to above. They joined workers on their daily abouts, attended meetings, and went on home visits with participants, they discussed what they saw as ‘good care’ in these specific situations. In addition to participant observation, we also interviewed service users, relatives, and other stakeholders such as the police, city council, social cooperatives and addiction-care services. As the research focused on the ethnographic work of following care professionals, one specific consequence is that these professionals’ perspectives on ‘good care’ are more pronounced than those of the other actors.

Informed consent was obtained for the interviews with service users. All material was anonymized, and no names or other persona details were collected.^6^ The fieldnotes and interviews collected during the fieldwork periods were transcribed, and then coded in Maxqda. The process of coding involved open and selective stages. In the open stage, to sharpen the focus of the research and to be sensitive to new questions, the material was analyzed during the fieldwork periods. It was then read multiple times and openly coded, parts being highlighted to identify recurring themes or patterns. This method of analysis made it possible for relationships and the use of spatial metaphors to emerge from the data as central themes. This analysis was discussed with the research team and a group of qualitative researchers to strengthen the coherence of the analysis. In the selective stage, we used a second round of analysis to identify cases or situations in which this relational approach was described or in which spatial metaphors were used.

Below, we describe daily care practices in one of the CMHCs in Trieste. Following the theoretical stand of empirical ethics, we have tried to analyze how good care takes shape in everyday practice. In the second part of the article, when our focus shifts towards spatial metaphors, we show how spatial understandings of care orient actors towards particular forms of good care.

## Findings

**I: working with and on relationships**

On one of my first days at the center, Mauro, one of the nurses, takes me and the interpreter to see a woman he has known for years. In the car on the way there he tells us that he sometimes jokes that he could divorce his wife, but not this patient. When we arrive at the apartment he rings the bell twice, telling us that this is how she knows it’s him. An elderly lady opens the door. When we enter, I notice that the walls are decorated with painted flowers. Mauro tells us to touch the paintings, explaining that they are made with toothpaste (mint for green) and he says with some pride that the woman is a true artist. He takes his time for a chat with her. We stay in a kitchen-cum-living room, where there are instant noodles and ready-made soup on the table. When he wants to give her depot medication, we go to another room, whose walls are decorated with small strips of insulating tape in the colors blue, yellow and red. It reminds me of Mondrian’s painting ‘Victory Boogie-Woogie’. The woman and Mauro join us again. The woman explains more about her art, but appears to be in her own world. She gives a long explanation about a connection with the trees and birds in the garden and the pots in her window, but with a logic none of us can follow.

Back in the car, Mauro explains a bit more about this woman’s situation. He describes it as a fragile equilibrium, but that they try hard to let her live independently, in her own way and in her own home. To make this possible, there are different forms of support, such as this morning’s check-ups and the medication from the mental health center. But she is also surrounded by a broader network. Over the years, her administrator and his wife, who became voluntary caregivers, do her shopping and check up on her. There is also social assistance to take care of her apartment, and frequent visits from her daughter, who lives in the city. As all these people are in regular contact with the center, the mental health professionals know how she is doing. Mauro also explains that her gas had been turned off for safety reasons (which explains the ready-made soup on the table) and that they have switched to depot medication, even though the mental health center prefers oral medication. This was necessary due to a crisis caused by the woman’s not taking medications about six months ago. When we’re driving back, I have the feeling that Mauro is proud of what they have managed to achieve with this woman, letting her live independently, in her own way.

A few months later, I hear from other nurses that she was in crisis again, and was staying at the center for a few months. The equilibrium is indeed fragile. (Fieldwork notes)

What can we learn from this story about what is perceived as good mental healthcare in Trieste? In this case description, ‘knowing’ one another is important in the relationship between Mauro and the woman. Mauro rings the bell twice, so she knows it is him. He takes time for us to get to know her a little, by pointing out the paintings, and describing her as a real artist and a unique person. This knowing goes together with being familiar: Mauro points out the personal and long-term nature of the relationship, by jokingly comparing their relationship to his marriage. On the trip there, he had already emphasized that he had known the woman throughout his whole working career.

If we take a step back and look beyond the traditional caregiver–patient relationship, it is clear that the care situation is also shaped by other actors. First, there are people: mental health workers, those from other services who provide support (supported housing); and her personal network (her daughter, and the administrator and his wife). There are also items and objects with specific qualities (her own apartment where she can make her toothpaste paintings without anyone protesting). There are forms and routines of support: the housekeeping, and the regular visits and medication by a nurse she knows well. There are also some restrictions: the gas has been cut off, and it has been decided to work with depot medication (which the mental health department sees as a less preferable option due to the invasive nature of its administration).

To use the words of nurse Mauro, all this is needed ‘to keep the equilibrium’. The word equilibrium-équilibrio’ in Italian—indicates being stable, a term often used in psychiatric care, but it is not the same. Being stable is a situation that can change back to being unstable. Equilibrium, in contrast, is more fragile: it can swing a bit, without collapsing, but caution and constant work are both required. ‘Hard work,’ Mauro says. ‘Maintaining the equilibrium’ is like a balancing act: skillful and playful at the same time.

What is also of interest here is what the director of the mental health care service in Trieste says about working to prevent a crisis:

It’s about creating a social surrounding that functions as a buffer – by providing housing, for instance, or relationships. If you asked a psychiatrist at an international meeting about preventing crisis, he would tell you that the most important thing is to be alert to early warning signs. And that is indeed of great importance. But what may be even more important is this kind of social engineering: working on the social determinants that create stability. Otherwise the circle maintains itself. (Interview with the Director of MH Dept in Trieste)

Here, the social surroundings are defined as an actor in care situations. Their role is to function as an instrument that makes it possible to work in the community without the facility of a closed ward or places for long-term admission. In crisis situations, as Mezzina and Johnson indicate (2008), the social network is involved as much as possible both in building a relationship and in maintaining connections with the outside world. If the network is weak or lacking, it is the task of the mental health care service to create one by working on what is called ‘social engineering’, i.e., by creating or maintaining housing, jobs, and social relationships. The importance of this social engineering is not purely instrumental: it is also a way of working on the ideal of social inclusion (Mezzina 2016).

### Care Collective

To understand the relational approach to community mental healthcare in Trieste, it is therefore necessary to be aware of the service users’ social surroundings and to examine the role of all those involved—not only service users and professionals, but also the other people and things involved in specific care situations. Winance (2010) has argued with regard to the analysis of care situations that it may be useful to speak of a care collective, as this will create the space in which it becomes visible that everybody involved has a role in providing care. In her analysis of the process of testing wheelchairs, Winance convincingly shows that a care collective is composed of different elements, people and things. In striving for ‘good’ care, she argues, everybody in the collective plays a part in negotiating what this good is. Seen from this viewpoint, the example of Mauro’s home visit shows how the care situation we observed was shaped both by human actors (the nurse, homecare, the family) and by non-human actors (depot medication, the fact the gas had been cut off, and the privacy of the old lady’s own apartment). This is not all: the actors themselves are ‘shaped’ by the relationships in the care collective, which define the way Mauro and the old lady relate to each other, the topics they discuss, the things Mauro observes, the medication that is given, and the food she is able to prepare in her own home.

### Negotiating Goods

When I was taken on this home visit, I had the sense that Mauro was proud of the situation, of what they had achieved. That is not to say that the situation was easy or self-evident. The reality was complex, and the old lady was impaired in many ways by her disabilities. As Winance (2010) points out, the ‘good’ in care situations is not a perfect situation in absolute terms. She sees this as inherent to care situations, as a collective is all about negotiating and dealing with the resources available: “the good is an arrangement of people and things that is a compromise, allowing life together and allowing motion and emotion for all those involved in the collective” (Ibid: 109). In this case, for instance, this may mean that an intervention that is labeled as less desirable—such as depot medication—is adapted in order to keep this equilibrium. A team leader commented on this in an interview:

“The way we normally function is to create continuity in the creation of the relationship, also with the family and others: it allows you to feel what’s going on. To create this continuity, we need signals in the context of a person: family, neighbors, school. So actually we need a good connection with others in the context who receive these signals. If this connection is good, it’s very easy to work on prevention.” She stresses that a good relationship is an open one, not a formal one. “It’s about calling each other, asking what’s going on – it’s about a trustful connection.” (Interview with a team leader of a CMHC in Trieste)

This gives us some more ideas about what is seen as important in shaping such a care collective: the relationships between the people involved are informal and trustful it is a relationship you can depend on. The aim is to achieve continuity in them. To achieve this, and to sustain relationships with the service users and others, workers should know what is going on. And, by knowing it, the ‘we’ in this case—i.e., the workers in the team—presumably know what kind of support people need. For this to be possible, the workers thus need the network of (family, neighbors, schools etc.) around somebody. As a ‘good’ professional does not—and cannot—act alone, this sometimes means that what is good is negotiable, not only with the service user, but also with other caregivers:

On one of the visits I make with a young psychiatrist, she visits a group house where a girl lives. At the family’s request, the psychiatrist had reduced sedative medication. The team is now having a meeting about the current situation, which they describe as *una crisi brutta* – a severe crisis. The girl is often aggressive towards the care team, herself and group members. The caregivers would like the psychiatrist to prescribe sedatives. The meeting takes over an hour, with the group house team members referring repeatedly to all the problems they have in her daily care. In the end the psychiatrist decides not to prescribe sedatives, but to switch to other antipsychotics. In the car on the way back, she reflects on the meeting: “For me it was searching. Persuading is also persuading the team: showing that you’re there for them and persuading them that sedatives are not a solution.” But she stresses the importance of listening to the team and their problems. (Fieldwork notes)

Closer examination of the role of the social network reveals a number of other elements that stand out. First, service users need a network around them that is broader than the mental health services. For a network to function as a buffer, the people in it should have a close relationship with the center, and should work together, signaling if something is changing and extra help or support is needed. When asked what he did when somebody refused care, a rehabilitation worker reflected that he would negotiate and persuade, engage in a dialogue and visit more often. But he would also contact neighbors and family. This sometimes gave him a new and different way of entering into a relationship. Being part of this care collective is not strictly voluntary. To make it function, each participant has a responsibility. The responsibility of the mental health care service is articulated as being accessible and accountable, and as responding quickly to a crisis when necessary (Mezzina 2016). One way to achieve this can be seen in the option patients and others have of walking into the center and being able to talk to a nurse, all without a waiting list. But other parts of the collective, like family, have a responsibility as well:

The next case in the team meeting raises a lot of emotion. The team worries about the time a young woman spends between school and home, as the mother does not watch over her and overestimates her daughter’s capabilities. Neither does the mother show up for appointments at the center. In a meeting later that week, the psychiatrist tells that she called in and confronted the mother with the fact that she was not sticking to the agreements that had been made with the team. They all stress the importance of the mother being involved, of her taking her responsibility. (Fieldwork notes)

As one of the ideals is that service users should be embedded in a social network in order to be provided with good care, it is not only family, neighbors and friends that are seen as necessary elements in a care collective: so, too, are work, school or housing. For instance; we observed that nurses immediately contacted the university to reschedule exams for a young man who was brought to the center in a crisis. Again, this network or collective is not only an ideal, but also an instrument; as well as being a way of working on the ideal of moving people ‘towards society’, it is a way of dealing with crisis without the facilities of closed wards. If there is no network, or if a network is in decline, it is seen as a problem, as there is—or soon will be—none to buffer a crisis.

It is important to note that in Italy cultural expectations around family relationships differ from ideas about family life in more northern European countries like the Netherlands. This could make it more easy to create stable networks. But despite the importance of family in the Italian culture, the role of the family in the care collective is not always taken for granted. In other cases we encountered during the fieldwork, the role of the family was problematized, for instance in the case of two young men who both had a ‘symbiotic’ relation with the mother in the opinion of the care professionals. In these cases one of the goals of the care was to provide independent housing for these boys, for instance in a shared apartment with other service users. Another way of ‘managing’ family relations was by trying to schedule the telephone calls of a mother to her sun (only in the evening).

### Role of the Professional

If one of the aims of care is to build and sustain relations, than this has an impact of the task scope of care workers: it is less about reducing symptoms as it is about creating and sustaining these relations. Professional therefore has to be involved in several domains of service users’ lives beyond that of their mental health:

It’s Saturday afternoon, just after lunch. Some of the care professionals and service users go for a smoke on the small balcony. This morning I joined the psychiatrist on her work. She had four meetings with service users and their families each of which took about an hour. None of the service users seemed to be urgently in need of something. On the balcony, me and the psychiatrist are reflecting on one of the meetings. It was with a mother and brother of a service user, mainly because the brother was worried that the burden of caring for his sick brother was becoming too much for his mother. The brother in the center’s care was in his forties, and still living at home.

As he was also financially dependent on his mother, in the meeting his financial situation was discussed. I was surprised by the time the psychiatrist had devoted to arranging social benefits for him: she had looked up the options on the internet and made an appointment to go to the municipality with him. I commented that, in the Netherlands, this would not be part of a normal Saturday morning routine for a psychiatrist on most of the mental health care teams I knew. I asked if a social worker wasn’t supposed to arrange such things. She stated that it was important to do it herself, because, “as a psychiatrist, you’re dealing with a service users’ whole life”. I commented that the importance of addressing all life domains is also stressed in the Netherlands, but that a psychiatrist focuses mainly on treatment and medication, a social worker on administration, and a nurse on daily assistance. Her reply: “It’s not therapeutic to work like that. As a psychiatrist you need to be involved in all life domains to understand what’s going on, and to build on a relationship.” (Fieldwork notes)

Once again, the focus lies on the ability to build relationships—in this case the role of the professional (the psychiatrist). The psychiatrist claims it is important to be involved with a service user’s ‘whole life’, and thus that division of labor is not regarded as a therapeutic way of working.

Above, we outlined how building relationships lies at the heart of the ‘goods’ the actors in care situations strive for. Good care is aimed at building and maintaining a social network around people. We argued that, to understand this approach, it is helpful to speak of a care collective that includes people as well as things, techniques and routines. The next thing that stood out when analyzing this relational approach was that ideas of good care were often addressed using spatial metaphors that indicated whether or not people were in the ‘right place’. These metaphors were sometimes about a material place, but also concerted social places—the way people related to others.

## Notions of Space

Questions of space and (mental) healthcare are addressed in the literature on human geography (Krause, Parkin and Alex 2014). To indicate the importance of place in health care, both physical as cultural, Gesler (1992) coined the term ‘therapeutic landscape’. As social spaces are shaped by social interactions and the material environment, other authors point out that social spaces can be understood as both physical and social at the same time (Pols 2016; Wood, et al. 2013; Gesler 1992; Doroud, Fossey and Fortune 2018; Smith, et al. 2015; Ootes et al. 2013; Ootes 2009). We do not only consider references to physical locations as spatial notions. In our fieldwork we noticed that spatial metaphors like ‘inclusion’ or ‘isolation’ were also frequently used. These terms do not solely refer to a physical place. Instead, they reflect particular ideas about how people (do or do not) interrelate. To understand how different forms of spatiality are oriented towards particular forms of good care, it can be helpful to use two concepts introduced by Mol and Law who explore different types of spatiality (Mol and Law 1994) Therefore we use their concept of a *network space* to make a contrast with *regional space.*

In a regional space, there is a vision of here and there, each being located on its own side of a boundary. This also makes it possible for there to be an “inside” and an “outside” (p. 647). In contrast, a network space links objects in space through the relationships between them, rather than through a regional ordering (p. 649). Network space is not about here or there, but about the way elements—such as people, machine and gestures—hang together (ibid). Using ethnographic data, we show how, in Trieste, these two different forms of spatiality are connected with specific ideas about good care. This can be illustrated by the following discussion about where people eat lunch:

When I enter the central room that is used both for team meetings and for lunch, the table is already set. Some service users are seated, others walk in. There is one large table for ten people, with some smaller ones around it. I take a place opposite a man in the corner. We chat a bit and it turns out that he was born in the Netherlands, but has forgotten most of the language: we practice ‘goedemorgen’ together. Then a woman in a white uniform comes in with a lunch trolley.

One man gets his food a bit earlier than the others. He is moving around a lot, mumbling to himself. He stands out because of his outfit. He is wearing only cycling shorts, is covered in tattoos and has lots of bracelets and strips of cotton around one arm. I often encounter this man near the railway station, talking to himself. His feet, injured and malformed, remind me of the ‘walking feet’ I remember from the homeless shelter where I used to work in Amsterdam. Nobody seems to mind him. He eats his food at enormous speed and then leaves the room.

After choosing between two menu options, the others get a primo consisting of pasta or rice with vegetables, and a secondo with meat – in today’s case, chicken. As the plates have already been prepared in the kitchen, there is no choice regarding the amount of food or anything else. Nurse Mauro had explained sometime earlier that the hygiene rules do not allow service users to cook in the health center or to help distributing the meals. Afterwards, some service users help clear away the dishes. I try to do so, too, but am told not to, since I am a guest. (Fieldwork notes)

On my first visits to the center, the social and material organization of the lunch was causing lot of discussions and team meetings. Recently the central department has planned to reallocate the places where people had their lunch. Rather than giving out meals in the center—where a group of service users had lunch every day—the idea is to create other options, such as organizing a cooking project in a group apartment, or eating at a local restaurant. One reason for this change had been an analysis of the situation at the centers showing that, for a small number of service users, the centers had become the only point of reference. This applied particularly to men aged between 45 and 55 whose condition was chronic. The head of the rehabilitation service explained that many things, including work programs and training, had already been tried for this group in the past, but without success. She stated that the idea behind the decision to relocate lunch was to reallocate activities and resources from the center to the community:

About 10 years ago we started various projects, all of them intended to make a move towards society. (…). The idea is to strengthen links with the community by creating new projects around distributing meals. These links are important, as they help us work on social cohesion. That is of importance, as it makes it easier to manage problems. (Interview with the head of the rehabilitation service)

But not everyone at the center agrees with this vision. A nurse explains to me that the center is a point of contact, a place for meeting each other. As lunch provides a means for staying in contact with some of the users—especially those who are more difficult to reach—it is an instrument that helps to check how people are doing and also provides an opportunity to work on relationships with them. On the other hand, most mental health professionals acknowledge that some users depended on the center. For those who had few contacts outside, it had become a ‘mini hospital’, without much contact outside the care facility.

If we look at the different spatiality’s used in the discussion about where to organize lunch—there are two notions of spatiality, each echoing different ideas of good care, and each in tension with the other. First, there is regional space—physical space; the boundary between ‘here’ and ‘there’—which is present in the discussion about the location of the lunch. In this notion of spatiality, lunch is an obstacle to an important ideal of good care: the ideal that service users should not live in the ‘separate world of psychiatry’, but should instead be part of the community. As lunch binds people to the center, the rather institutional mode of distributing the food described above—with fixed portions, and where helping was not allowed – does not contribute to the ideal of moving people out of ‘the institution’ into ‘the community’. Moving lunch from a perspective of regional space echoes one aspect of the ideal behind ‘deinstitutionalization’, that patients should be made into citizens by enabling people to take part in the community by moving the place where care is provided. Following Ootes (Ootes et al. 2013; Ootes 2009), who argues that notions of space are related to notions of citizenship, we could argue that relocating lunch is also a way of working towards this idea of good care. It goes together with the hope that organizing lunch at various other locations will extend service users’ abilities to participate (in the community), for instance by learning how to cook.

Second, there is the spatial metaphor of the network, a social space that refers to relations between people and things and how they hang together. In this network space, the service user is the center of a network or web of people and things that functions best when its participants have close relationships. This network space echoes an important idea about the professionals’ provision of good care (see above), where the aim is to stay in a close relationship with service users and to build a network. In this network space, lunch at the center is an important instrument for achieving both at the same time: as people visit the center every day to eat, they meet other people and the care professionals are offered a means of building and maintaining relationships.

The discussion about lunch and the types of spatiality used, direct us towards different ideas of good care that are related to different aspects of the process of deinstitutionalization. From the perspective of regional space—which echoes the ideal of deinstitutionalization in the sense that it is about moving people ‘outside’—moving the lunch is seen as a way of strengthening the link with the community, and therefore as ‘good’. From a network perspective in which deinstitutionalization is more about the relations between actors and the possibilities abilities to participate in the community, things are more complicated: nurses drew attention to the risk that the relationships with care workers and service users would become weaker if the daily lunch visits to the center disappeared, and that it would then become more difficult to maintain strong relationships and to maintain the network in which, ideally, service users were embedded. The example of the lunch makes clear that deinstitutionalization is more than a shift from the hospital to the community in practice it asks for finding ways of doing things, articulating good care. By doing this, relationships and social spaces are shaped and reshaped.

### Living Independently

Back to the house of the lady who made the toothpaste paintings for another example of how spatial configurations project ideals about good care, but in complex ways. In the philosophy of deinstitutionalization whereby hospital beds are replaced by care in the community, living independently is seen as an aim to strive for. This is reflected in the organization of care described above. The center is located in the community, and to work in such way that there is no need to transfer someone to an institution, the thresholds are low: care is provided where people live. But in daily care practices, things are more complex than a chronological sequence from a regional spatiality (the move from the hospital towards the community) towards a network spatiality (the idea of a service user who arrives in the community as a relational being and becomes embedded in a network), and the different ideas about good care that are reflected in these spatial metaphors.

How it is more complicated becomes clear in situations were living independently is not always seen as ‘good’ by those involved. During our fieldwork, for instance, there was much discussion amongst nurses about a man in the center’s care who was strongly alcohol dependent. Before being admitted to the center he had caused a lot of problems by involving other service users in his drinking. Now he stayed in his own home drinking, and it was difficult for the care workers to contact him. In a telephone meeting with his administrator, a nurse described the situation as a *micro*-*manicomio. Manicomio*—‘asylum’ or ‘madhouse’—is the term that was often used during the reforms of the 1970s to stress that psychiatric hospitals were what Basaglia had termed ‘total institutions’ that cut people off from society and deprived them of their individuality. Care workers also used this term to describe situations in which service users lived independently and were simultaneously socially isolated. The use of the term *micro*-*manicomio* echoes a relational-spatial perspective: people should not be socially isolated, but part of the community. One way of achieving this was by creating and maintaining care collectives. So the deinstitutionalizing principle of living independently in the community is not therefore labeled as ‘the good’ in all situations. It is conditional: the ideal of living independently in the community exists next to the ideal of a person as a relational being who is also embedded in social networks. A good patient is in a relationship with the world around him: making steps outside, possibly in a job, or involved in other daily routines:

The challenge is that users find their way in the normal world, not the shielded world of psychiatry such as a hospital. We can never be the only bridge to social reality, we need more bridges. It’s about normalization. (Interview with the Director of MH Dept in Trieste)

The use of the word *micro*-*manicomio* also indicates the importance of the sociality of space in providing care. It is not only about the regional place where somebody lives—a person’s own apartment. It is also about the network space, which we defined as both physical and social. The word marks a shift from isolation and a parallel world towards spatial network configurations in which people are embedded in relationships, and ‘in the normal world’. There is a realization that it takes time to build these relationships, and that it is the service user who determines the pace. Again and again, professionals in team meetings stressed that things should be done *piano piano*—step by step.

## Conclusion

In this article we have asked how mental health care can be shaped in the community, and how it can be thought of as good care. As a practice to learn from, we chose the community mental health care services in Trieste due to the unique and radical process of deinstitutionalization the city has gone through. What could their daily practice of care teach us about the specificities of and notions of good care embedded in everyday community care? We suggest that our analytical method may support an understanding of what is at stake in everyday care practice, here, specifically, in relation to the spatial metaphors implicit in these practices.

Firstly, our analysis of daily care practices developed what we may call a ‘radical relational approach’ (Pols 2014; Driessen 2019). This term takes into account that relationality comes in many forms, including things, activities and words. The house visit to the ‘tooth paste artist’ made visible that to understand care practices, it is of importance to widen our scope from the dyadic relationship between care professional and patient, to include a broader care collective of actors that includes material things, buildings and spaces (Winance 2010).

This analysis showed us how professionals enact good care. They describe situations as ‘good’ when service users are part of a care collective. These relational arrangements foregrounded the sociality built as network space: a *good* situation is a situation in which somebody is in contact with others, participates in meaningful activities and infrastructures, and is integrated into society by being part of a socio-material network. As well as being an ideal to strive for, Trieste’s network approach to mental health care is also the instrument for bringing about good care, as creating a network is used as a way of preventing crisis, without relying too much on (scarce) clinical facilities. A good professional in Trieste works on building and maintaining long-term relationships, not only with the service users, but, also with the other nodes in the network that include work, school, places to go to and family. In this perspective good mental healthcare is not primarily about the reduction of symptoms. Good care is about sustaining and building networks within which individuals may live as well as they can.

This focus on the network has some practical implications: it means that service providers have a specific understanding of what it means to be a mental health professional, whose central competence is to build relationships. A consequence of this radical relational perspective is that it enables health professionals to interfere, before psychiatric symptoms may emerge or increase. More interestingly, it leads to a practice of small, daily interferences and adjustments to keep up the network. An example: in case of a hospitalization not only family is involved, but the professionals also immediately make an effort to manage relations with employers or school, as to smoothen the return. Another example is the arranging of social benefits by a psychiatrist, as our example showed. To work and maintain these relations in the network is seen as much as part of the job as the reduction of symptoms is. This does, however, demands a certain kind of de-specialization, because all service providers, invest in relationships with their clients and their network. Another consequence is that mental healthcare professionals are present for a long time in the lives of service users. This can be at odds with the ideal of normalization that is present in mental healthcare as well. Articulating the Trieste’s relational approach to mental health care resonates with criticism on dominant biomedical views in which mental health problems are mainly presented as disorders that can be diagnosed in the individual. These need treatment from medical specialists who may apply evidence based interventions (see for this criticism: Van Os et al. 2019). The relational approach decenters the individual by focusing on how these patients do or do not relate to other (social and material) actors in their network.

Secondly, our study shows how deinstitutionalization is not a mere organizational change signified by a reduction of beds or a number of service users moved out of psychiatric institutions. The idea of successful deinstitutionalization relates to a regional understanding of space. An understanding of the relational aspects of care shows that reducing beds and providing housing does not automatically lead to social contacts and becoming part of a community (Pols 2016).^7^ The relational approach as practiced in Trieste mental healthcare teaches what may be done once the hospital is closed and regional moves have finished.

The analysis of spatial metaphors and how they project ideas about good community care makes visible that deinstitutionalization is not only a chronological sequence that moves from a regional spatiality towards a network spatiality. The discussion about where to provide lunch shows the regional metaphors are still active. This created tension between different understanding of what is good care: while providing lunch at the center supported the creation of relationships and a network between and around service users, moving it to restaurants in the community might serve a different case: the ideal of moving care towards different relationships with different people. The discussion about lunch shows that in daily practice, finding ways of providing good care implies a juggling of different approaches and metaphors. Articulating the metaphors and the goods that are at stake teaches how good care may practically take shape, and how it differs from other practices and their embedded goods. This may help us reflect on what form of good care achieves what in different situations.

## Footnotes

1 We perceive deinstitutionalization first as a form of regional spatiality in which care is transferred from the hospital into the community. But the process of deinstitutionalization also involves the daily care practice that is then implemented in the community. In this, we argue, social notions of space are important.

2 For instance, not all Italian regions have a 24-h community-based service (Mezzina 2016). Such uneven coverage is underlain by differences in the availability of resources for mental health care (Barbui and Tansella 2008). Other authors have indicated political factors. For example, De Vito (2015) states that Italian psychiatric reform in the decades following the implementation of Law no. 180 faced fierce opposition from conservatives, professionals, politicians and various organizations representing the relatives of psychiatric patients. He claims that the application of Law 180 was sabotaged more or less explicitly by the national government, which in some regions led to the virtual abandonment of chronic patients. See also Fioritti (2018).

3 In the article they distinguish a third form of spatiality, one in which social spaces behave like a fluid. This form is less relevant to the argument we wish to make here.

4 Central to this research was the question of what, when shaping its own process of deinstitutionalization, Dutch mental health care can learn from Trieste’s 40 years of experience of working in the community. For this project we spoke with stakeholders, operators, partners, and had interviews with service users and their networks (Muusse and van Rooijen 2015).

5 Next to the use of Italian, conversations between the researcher and care workers/service users were conducted in English as well. In this article we try use the Italian term for keywords, but due to this double use of languages, this was not always possible.

6 The METC from VU University (FWA00017598) has declared that the Medical Research Involving Human Subjects Act (WMO) does not apply to the study.

7 Pols (2006, 2016) uses the notion of ‘relational citizenship’ to signify becoming part of a network as a way to become a citizen.

## References

[CR1] Barbui Corrado, Tansella Michele (2008). Thirtieth Birthday of the Italian Psychiatric Reform: Research for Identifying Its Active Ingredients is Urgently Needed. Journal of Epidemiology and Community Health.

[CR2] Bennett D. (1985). The Changing Pattern of Mental Health Care in Trieste. International Journal of Mental Health.

[CR3] Burti Lorenzo (2001). Italian Psychiatric Reform 20 Plus Years After. Acta Psychiatrica Scandinavica.

[CR4] Chow Winnie, Ajaz Ali, Priebe Stefan (2018). What Drives Changes in Institutionalised Mental Health Care? A Qualitative Study of the Perspectives of Professional Experts. Social Psychiatry and Psychiatric Epidemiology.

[CR5] Chow Winnie, Priebe Stefan (2013). Understanding Psychiatric Institutionalization: A Conceptual Review. BMC Psychiatry.

[CR6] De Vito, Christian. 2015. Liminoids, Hegemony and Transfers in the Liminal Experiences in Italian Psychiatry, 1960s–1980s. In Ausnahmezustaende. Entgrenzungen und Regulierungen in Europa waehrend des Kalten Krieges. Cornelia Rauh und Dirk Schumann, hrsg., pp. 236–252. Goettingen: Wallstein Verlag.

[CR7] Doroud Nastaran, Fossey Ellie, Fortune Tracy (2018). Place for Being, Doing, Becoming and Belonging: A Meta-synthesis Exploring the Role of Place in Mental Health Recovery. Health and Place.

[CR8] Driessen, Annelieke 2019 A Good Life with Dementia, Ethnographic Articulations of Everyday Life and Care in Dutch Nursing Homes, Thesis. Amsterdam: Universiteit van Amsterdam.

[CR9] Fioritti Angelo (2018). Is Freedom (Still) Therapy? The 40th Anniversary of the Italian Mental Health Care Reform. Epidemiology and Psychiatric Sciences.

[CR10] Foot John (2014). Franco Basaglia and the Radical Psychiatry Movement in Italy, 1961–78. Critical and Radical Social Work.

[CR11] Foot John (2015). The Man Who Closed the Asylums: Franco Basaglia and the Revolution in Mental Health Care.

[CR12] Foot John (2015). Photography and Radical Psychiatry in Italy in the 1960s. The Case of the Photobook Morire di Classe (1969). History of Psychiatry.

[CR14] Gesler Wil (1992). Therapeutic Landscapes: Medical Issues in Light of the New Cultural Geography. Social Science and Medicine.

[CR15] Goffman Erving (1968). Asylums: Essays on the Social Situation of Mental Patients and Other Inmates.

[CR16] Henckes Nicolas (2011). Reforming Psychiatric Institutions in the Mid-twentieth Century: A Framework for Analysis. History of Psychiatry.

[CR17] Krause Kristine, Parkin David, Alex Gabi (2014). Turning Therapies: Placing Medical Diversity. Medical Anthropology.

[CR19] Mezzina Roberto (2016). Creating Mental Health Services Without Exclusion or Restraint But with Open Doors Trieste, Italy. L’Information psychiatrique.

[CR20] Mezzina Roberto (2014). Community Mental Health Care in Trieste and Beyond. An “Open Door-No-Restraint” System of Care for Recovery and Citizenship. The Journal of Nervous and Mental Disease.

[CR21] Mezzina Roberto, Johnson Sonia, Johnson Sonia, Needle Justin, Bindman Jonathan P., Thornicroft Graham (2008). Home Treatment and ‘hospitality’ Within a Comprehensive Community Mental Health
Centre. Crisis Resolution and Home Treatment in Mental Health.

[CR22] Mol Annemarie, Moser Ingunn, Pols Jeannette, Mol Annemarie, Moser Ingunn, Pols Jeannette (2010). Care: Putting Practice into Theory. Care in Practice: On Tinkering in Clinics, Homes and Farms.

[CR24] Mol Annemarie, Law John (1994). Regions, Networks and Fluids: Anaemia and Social Topology. Social Studies of Science.

[CR25] Muusse Christien, van Rooijen Sonja (2015). Freedom First, Een onderzoek naar de ervaringen met wijkgerichte ggz-zorg in Triëst, Italië, en de betekenis hiervan voor Nederland.

[CR26] Novella Enric J. (2010). Mental Health Care in the Aftermath of Deinstitutionalization: A Retrospective and Prospective View. Health Care Analysis.

[CR27] Ootes Sabine (2013). Where is the Citizen? Comparing Civic Spaces in Long-Term Mental Healthcare. Health and Place.

[CR28] Ootes, Sabine 2009 Being in Place: Citizenship in Long-Term Mental Healthcare. PhD Thesis.

[CR29] van Os Jim (2019). The Evidence-Based Group-Level Symptom-Reduction Model as the Organizing Principle for Mental Health Care: Time for Change?. World Psychiatry.

[CR30] Pols Jeannette (2016). Analyzing Social Spaces: Relational Citizenship for Patients Leaving Mental Health Care Institutions. Medical Anthropology.

[CR31] Pols Jeannette (2015). Towards an Empirical Ethics in Care: Relations with Technologies in Health Care. Medicine, Health Care and Philosophy.

[CR32] Pols Jeannette, Olthuis Gert, Kohlen Helen, Heier Jorma (2014). Radical Relationality. Epistemology in Care and Care Ethics for Research. Moral Boundaries Redrawn: The Significance of Joan Tronto’s Argument for Political Theory, Ethics of Care as Argument for Political Theory, Ethics of Care.

[CR33] Portacolone Elena (2015). A Tale of Two Cities: The Exploration of the Trieste Public Psychiatry Model in San Francisco Culture. Medicine and Psychiatry.

[CR34] Ridente Pina, Mezzina Roberto (2016). From Residential Facilities to Supported Housing: The Personal Health Budget Model as a Form of Coproduction. International Journal of Mental Health.

[CR35] Shen Gordon, Snowden Lonnie (2014). Institutionalization of Deinstitutionalization: A Cross-National Analysis of Mental Health System Reform. International Journal of Mental Health Systems.

[CR36] Smith Bikki T. (2015). Rebuilding Lives and Identities: The Role of Place in Recovery Among Persons with Complex Needs. Health and Place.

[CR37] Willems Dick, Pols Jeannette (2010). Goodness! The Empirical Turn in Health Care Ethics. Medische antropologie.

[CR38] Winance Myriam, Mol Annemarie, Moser Ingunn, Pols Jeannette (2010). Care and Disability Practices of Experimenting, Tinkering with and Arranging People and Technical Aids. Clinics, Homes and Farms.

[CR39] Wood Victoria (2013). Creating ‘therapeutic landscapes’ for Mental Health Carers in Inpatient Settings: A Dynamic Perspective on Permeability and Inclusivity. Social Science and Medicine.

